# Drug Discovery and Development of Novel Therapeutics for Inhibiting TMAO in Models of Atherosclerosis and Diabetes

**DOI:** 10.3389/fphys.2020.567899

**Published:** 2020-10-29

**Authors:** Ian Steinke, Nila Ghanei, Manoj Govindarajulu, Sieun Yoo, Juming Zhong, Rajesh H. Amin

**Affiliations:** ^1^Drug Discovery and Development, Auburn University, Auburn, AL, United States; ^2^Department of Anatomy, Physiology and Pharmacology, Auburn University, Auburn, AL, United States

**Keywords:** FMO3, TMA, CVD, T2D, TMAO, dysbiosis, atherosclerosis, microbiome

## Abstract

Diabetes mellitus exists as a comorbidity with congestive heart failure (CHF). However, the exact molecular signaling mechanism linking CHF as the major form of mortality from diabetes remains unknown. Type 2 diabetic patients display abnormally high levels of metabolic products associated with gut dysbiosis. One such metabolite, trimethylamine N-oxide (TMAO), has been observed to be directly related with increased incidence of cardiovascular diseases (CVD) in human patients. TMAO a gut-liver metabolite, comes from the metabolic degenerative product trimethylamine (TMA) that is produced from gut microbial metabolism. Elevated levels of TMAO in diabetics and obese patients are observed to have a direct correlation with increased risk for major adverse cardiovascular events. The pro-atherogenic effect of TMAO is attributed to enhancing inflammatory pathways with cholesterol and bile acid dysregulation, promoting foam cell formation. Recent studies have revealed several potential therapeutic strategies for reducing TMAO levels and will be the central focus for the current review. However, few have focused on developing rational drug therapeutics and may be due to the gaps in knowledge for understanding the mechanism by which microbial TMA producing enzymes and hepatic flavin-containing monoxygenase (FMO) can work together in preventing elevation of TMAO levels. Therefore, it is critical to understand the advantages of developing a novel rational drug design strategy that manipulates FMO production of TMAO and TMA production by microbial enzymes. This review will focus on the inspection of FMO manipulation, as well as gut microbiota dysbiosis and its influence on metabolic disorders including cardiovascular disease and describe novel potential pharmacological therapeutic development.

## Introduction

Heart disease is the leading cause of death in the United States today. Furthermore, diabetic patients are twice as likely to develop cardiovascular disease (CVD). Most recent reports on diabetes mellitus from the Center for Disease Control and Prevention estimate that 9.1% of the population in the United States of adults 18 years and older have been diagnosed, where type 2 diabetes (T2D) mellitus is being attributed to approximately 95% of these cases (Centers for Disease Control and Prevention, 2018).

In patients with diabetes mellitus, primarily T2D, atherosclerotic CVD predominates as the leading cause of mortality (Booth et al., 2006; Wang et al., 2016). CVD is characterized by inflammation induced atherosclerotic complications, resulting from an increase of lipid particles to the endothelial cells causing foam cell and the resultant plaque formation (Libby, 2006). T2D develops out of metabolic disorder, characterized by insulin resistance and reduced insulin secretion leading to chronically elevated blood glucose levels.

Other comorbidities associated with T2D are hypertension, dyslipidemia, and renal insufficiency (Iglay et al., 2016). Further increasing CVD pathology are T2D related risk factors that include decreased high-density lipoprotein (HDL) cholesterol, increased low-density lipoprotein (LDL) cholesterol, hyperlipidemia, hyperinsulinemia, and an increase of reactive oxygen species (ROS) in the blood (Ding and Triggle, 2005).

As T2D progresses, hyperinsulinemia, hyperglycemia, and oxidized LDL culminates in endothelial cell dysfunction and apoptosis (Ding and Triggle, 2005). This decrease in the proper functioning of endothelial cell walls coupled with glycated hemoglobin causes a reduction in blood flow to the vasculature, increasing blood pressure and risk of ischemic damage (Hosseini-Beheshti and Grau, 2019). In the final progression of atherosclerosis, plaque formations become unstable and rupture, dislodging from the endothelial cell wall, resulting in myocardial infarction, and stroke (Bennett et al., 2016).

Complicating CVD in T2D patients is the common association of combined hyperlipidemia, a pro-atherogenic lipid disorder, characterized by high levels of LDL, triglycerides (TGs), and decreased levels of HDL (Athyros et al., 2002). Improper metabolic utilization of dietary fatty acids can cause increasingly higher levels of LDL and TGs in the blood, coupled with ROS, that can initiate or exacerbate plaque formation in the endothelial membrane (Bai et al., 2015).

Current pharmacological therapies for T2D, when in conjunction with CVD, attempt to reduce associated risks through tight glycemic control, dietary changes, and statin drugs. However, statistical evidence shows no significant correlation in lowering the risk of CVD with these treatments alone when considering T2D patients (Leon and Maddox, 2015). Even when intensive lifestyle changes are implemented and T2D symptoms are reduced, the cardiovascular outcome remains unchanged (Pi-Sunyer, 2014; Ferrannini and DeFronzo, 2015).

Conversely, therapies that seek to lower LDL via clearance upon upregulation of LDL receptors have shown statistical significance in cardiovascular risk reduction (Silverman et al., 2016). These findings show promise in the ability to reorganize the levels of HDL and LDL in circulation, yet many therapies are unable to improve energy regulation through glucose metabolism and insulin resistance in unison with cholesterol.

This review will examine different metabolic pathways involved in dysbiosis and efforts to ameliorate the downstream physiological consequences. In addition to, an examination of how modulating enzymatic activity directly involved with formation of the biomarker trimethylamine N-oxide (TMAO) may improve health outcomes in patients with CVD and/or T2D.

## Gut Microbiomes Role in the TMA/TMAO Pathway

Recent research has intensified in the area of the gut microbiome and its implication in various disease pathologies exhibiting the significant role bacteria play in metabolic homeostasis (Sung et al., 2017). There exists a vast array of bacteria in the human digestive system that can facilitate metabolism, immune health, and secondary energy scavenging based upon the host’s dietary intake (Simon and Gorbach, 1986). Due to the diversity and adaptability of the gut microbiome, changes in diet can quickly and dramatically influence the species of bacteria present; thus, altering the concentration and effect of bacterial metabolites that can influence the host metabolism (Arora and Bäckhed, 2016). Furthermore, the potential of the gut microbiome to metabolize dietary compounds is so profound that it equals that of the liver and is capable of being classified as an organ (Sommer and Bäckhed, 2013), demonstrating a propensity to influence host absorption of undesirable compounds. Bacteria respond quickly to dietary changes by an increase or decrease in the number of their species based upon the dietary source available (Rachel et al., 2015). Major changes in gut microbiome taxa are influenced most significantly by high carbohydrate and high protein diets but show no significant change in response to high lipid content (Holmes et al., 2017). These changes in the microbiota can have a dramatic impact on intestinal permeability and alter cardiovascular outcomes through inflammatory pathways (Sandek et al., 2012).

Along with microbial induced inflammatory effects, degradation of the mucosal membrane leads to infiltration of the epithelial membrane by other endotoxins, pro-inflammatory cytokines, and potentially harmful bacterial metabolites (Murphy et al., 2015). Crosstalk distribution of gut microbiota has been illustrated in the pathology of diabetes, obesity, CVD and metabolic syndrome (Lau and Vaziri, 2019). The major metabolites that are produced by beneficial gut microbiota are short chain fatty acids (SCFAs) such as butyrate, acetate, and propionate. These SCFAs are essential to maintain intestinal health, insulin, glucose, and lipid homeostasis (Lau and Vaziri, 2019). Recent research showed the proatherogenic contribution of microbial metabolites such as increased trimethylamine (TMA) and decreased SCFAs in CVD (Liu et al., 2019; Tang et al., 2019).

Moreover, structural and functional intestinal alterations arising from a lack of diversity in the microbiome have shown a correlation with increased heart failure related risk and incidence (Luedde et al., 2017). Marques et al. (2017) implied increases in phyla Bacteroidetes and Proteobacteria and decreases in phyla Firmicutes and Fusobacteria strains with changes in their related metabolites, like TMA to TMAO, with relation to chronic heart disease. Primarily two different species of bacteria (Firmicutes and Proteobacteria) have been identified as being responsible for the metabolism of choline to produce TMA (Velasquez et al., 2016).

In addition, there exists links between microbiota dysbiosis and vascular tone resulting in blood pressure regulation by regulating cardiac and renal early growth response 1 protein (Egr1) and the renin-angiotensin system (Marques et al., 2017). Marques and colleagues observed in apparent mineralocorticoid excess mouse models that were fed a high fiber diet with a SCFA acetate supplement, Egr1, markers of hypertension and heart failure, were decreased in comparison to these mice fed with a control diet (Marques et al., 2017). Egr1’s role of being a master regulator of kidney, renal fibrosis and regulation of the renin-angiotensin system further validates the role dysbiosis can have by propagating host inflammatory responses through dietary intake and reorganization of commensal bacteria. Along these lines, the dysbiosis of the gut microbiome may have significant impact through the different bacterial metabolites and production of SCFAs toward intestinal mucosal barrier protection and excretory pathways. To add to this SCFA propionate has been observed to modulate blood pressure via Gpr41, which is a SCFA receptor expressed in smooth muscle cells of small vessels, exhibiting a vasodilatory effect and influencing the secretion of renin in the modulation of blood pressure (Pluznick et al., 2013).

Wang et al. (2015) demonstrated that there is a positive correlation between the TMAO pathway and progression of heart failure. Treatment in mice with 3,3-dimethyl-1-butanol (DMB), a choline mimetic, displayed potential therapeutic effects in CVD via inhibition of microbial cleavage of choline consequently decreasing circulating levels of TMAO. DMB is found in balsamic vinegar, olive oil, grape seed oil, and red wines, and has been observed to decrease macrophage-foam cell formation and aortic root atherosclerotic lesion development through the inhibition of the bacterial enzyme TMA lyase CutC/D in ApoE knockout mice (Wang et al., 2015; Velasquez et al., 2016). Other inhibitors such as meldonium, an analog of carnitine, has also shown to display anti-atherosclerotic activity by preventing bacterial TMA formation via competitive inhibition of bacterial carnitine palmitoyltransferase-1 (CPT1) (Velasquez et al., 2016).

Trimethylamine N-oxide has been associated as an independent risk factor, aside from genetic predisposition and environmental factors, for CVD and atherosclerosis (Mohammadi et al., 2016). Kamo et al. (2017) illustrated that patients with HF have higher plasma levels of TMAO than control subjects and show a positive correlation with increased incidence of mortality. Foods such as meat, eggs, and salt-water fish contain high levels of specific TMA containing nutrients, i.e., phosphatidylcholine, choline, and carnitine. Recent research has revealed that inhibition of specific bacterial enzymes, containing TMA lyase, can attenuate the CVD (Craciun et al., 2014). TMA is known to be a toxic gaseous compound that causes corrosion and necrosis of mucosal membranes. TMA is also a known inhibitor of acetylcholine esterase, blocking the ability of cholinergic neurons to return to their resting potential. TMA is rapidly absorbed in the intestines and progresses into the liver where it is metabolized by human flavin-containing monooxygenase isoform-3 enzyme (FMO3) into TMAO where it is readily excreted in the urine (Janeiro et al., 2018). Despite TMA’s known toxic effect, it is TMAO that has become commonly accepted as a biomarker of CVD (Barrea et al., 2018). Higher levels of circulating TMAO is of primary concern since greater than 90% of dietary TMA is excreted in the urine through this metabolic form, as demonstrated in the case of 167 healthy individuals (Al-Waiz et al., 1987). Moreover, TMAO levels are associated with an increased risk of major cardiovascular events in patients with diabetes (Zhu et al., 2016). However controversy exists as to whether TMAO influences the progression of CVD disease or acts as a bystander of dysbiosis and pro-inflammatory pathways when examining CVD and T2D patients (Landfald et al., 2017; Lau and Vaziri, 2019). Non-lethal inhibition of bacteria in this pathway, has shown potential value as a therapeutic target. Mechanistically, dietary choline supplementation was shown to increase foam cell formation, promoting atherosclerotic complications in Apoe knockout mice (Wang et al., 2011). Furthermore, non-lethal inhibition of TMA lyase using DMB demonstrated a decrease in foam cell formation in Apoe knockout mice resulting in decreasing in circulating TMAO levels in the blood and cardiovascular complications (Wang et al., 2015). These results reinforce the viability of TMA lyase as a potential therapeutic target to alleviate symptoms related to thrombotic events and ischemic damage.

Trimethylamine N-oxide has been shown to induce endoplasmic reticulum (ER) stress through the elevation of ER-HSP70 isoform (Mohammadi et al., 2016), and by directly binding to ER stress kinase PERK whereby its activation lead to induction of FoxO1 (Chen et al., 2019). Other findings have observed that TMAO promotes foam cell formation by upregulating macrophage scavenger receptors, resulting in the development of atherosclerosis (Shih et al., 2019). SR-A1, and multiple scavenger receptors such as LOX-1, CD36, and SR-A1, contribute to the development of atherosclerosis by enhancing the uptake of cholesterol with lipoprotein modification. Wang et al. (2011) reported that increased TMAO levels enforced macrophages to accumulate cholesterol by inducing SR-A1 as a scavenger receptor. In addition, Mohammadi et al. (2016) showed that TMAO-induced upregulation of SR-A1 at mRNA and protein levels, contributed to the development of ER stress as well as lipid-laden macrophage activation in atherosclerosis (Hotamisligil, 2010).

**FIGURE 1:**
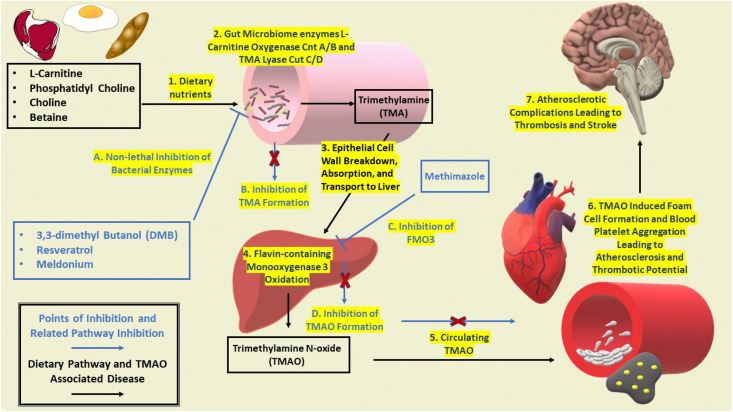
Crystallographic active sites of TMA lyase CutC/D and FMO3 with chemical structures of current known inhibitors. **(A)** Chemical structures of substrates and inhibitors of TMA lyase (Wang et al., 2015). **(B)** Choline bound to TMA lyase showing key binding interactions with active site amino acid residues that occur upon activation PDB 5AOU. **(C)** Chemical structures of dietary indole condensation products shown to be FMO3 inhibitors (Cashman et al., 1999). **(D)** Reactive enzymatic intermediates and key interactions with active site amino acid residues with TMA bound to FMO3 PDB 2GV8.

Elevated levels of TMAO have also been observed to inhibit mitochondrial electron transport, thus leading to mitochondrial uncoupling and ROS formation in vascular endothelial cells (Hotamisligil, 2010). In addition, increased plasma levels of TMAO positively correlate with the aggregation of blood platelet thrombosis (Tilg, 2016). Recent research with isotope-labeled phosphatidylcholine illustrated the direct correlation between increased serum TMAO and extent of coronary atherosclerotic plaque development (Tilg, 2016; Zhu et al., 2016). Figure 1 provides an overview for the microbiomes contribution to the TMA/TMAO pathway and eventual disease progression from dietary intake to atherosclerotic complications.

## Inhibition of TMA Lyase CutC/D

**FIGURE 2:**
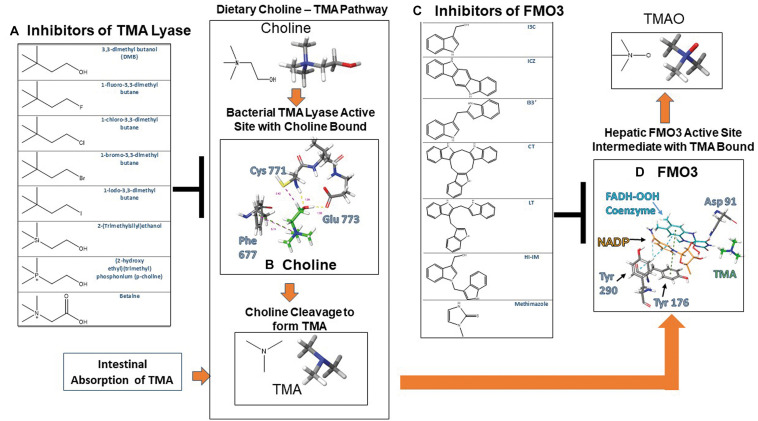
Development of dysbiosis and propagation of CVD and T2D with potential points of inhibition. Excess nutrients begin to influence dysbiosis of the gut microbiome to produce bacterial metabolite TMA. TMA lyase CutC/D as a main enzyme for inhibition as it is the most prominently expressed in bacterial communities (Rath et al., 2017). General progression of dysbiosis leads to further infiltration of unwanted bacterial by products leading to increased expression of FMO3 and elevated levels of TMAO. As circulating levels of TMAO begin to increase, pathologies related to CVD and T2D, reverse cholesterol transport is inhibited leading to lipid laden macrophages and increased blood platelet aggregation. Continued atherosclerotic complications then promote thrombotic events and stroke in the brain.

Trimethylamine lyase is a bacterial enzyme that is known to cleave dietary choline during an anaerobic process releasing TMA and acetyl aldehyde in the process. Recent reports have built DMB analogs to inhibit TMA formation by inhibiting CutC/D lyase. Figures 2A,B shows a representation for describing mechanistically how choline binds to CutC/D choline lyase. Choline is shown to interact with CutC/D via hydrogen bonding at the Cys 771 and Glu 773 sites of CutC/D, along with aromatic hydrogen bonds to Phe 677. When choline binds to TMA lyase, the active site residues engulf choline, whereby choline can stabilize the active site allowing the enzymatic reaction to proceed. Mechanistically the aromatic hydrogen bonding of the quaternary amine in choline with Phe 677 provides additional stabilization of the active site and provides improved chemical reactivity. In addition, TMA is formed by the Cys 771 promoting cleavage of choline through a radical exchange with the alpha carbon to form alcohol in the formation of acetaldehyde and TMA (Bodea et al., 2016).

Another study has shown that mechanistically DMB prevents the catalytic activity required from the substrate choline’s quaternary ammonium motif, thus providing a structure that can competitively bind but not exhibit reactivity, thus effectively inhibiting choline from being cleaved to TMA as illustrated in Figure 1 (Craciun et al., 2014). These findings are highly significant for development of novel compounds to prevent atherosclerosis in patients with western diets, as non-lethal inhibition promotes reorganization of the gut microbiome without drastically reducing the numbers of beneficial bacteria.

Trimethylamine lyase is also of important to consider, as strictly inhibiting FMO3, can lead to an increase in circulating TMA levels causing an unpleasant “fishy” smell in a condition that is known as Trimethylaminuria (Mitchell and Smith, 2001). To avoid this consequence and promote reorganization of the gut microbiome non-lethal inhibition of TMA lyase must be factored into the drug design of any potential therapeutics.

## Inhibition of FMO3

Of the major oxidative drug metabolizing enzymes in the body the Cytochrome P-450 (CYP450) family exists as the most prominently expressed, existing in over fifty isoforms, class of enzymes for first step biotransformation of xenobiotic compounds (Srinivas, 2017). More recent research has begun to shed light on the importance of another major oxidative drug metabolizing class of enzymes known as flavin-containing monooxygenases (FMO). FMO’s have distinct differences in function and substrate activity compared to the CYP450 class of enzymes resulting in different disease pathologies that may be accompanied with FMO expression or inhibition (Cashman and Zhang, 2006). FMO’s exist in five isoforms, with a sixth FMO existing as a pseudogene, this family of genes are conserved across all phyla and are characterized by their unique structural and functional differences (Ziegler, 1993).

Functional characterization of FMO3 is best understood when examining the polymorphisms that are commonly associated with the enzyme. Single nucleotide changes can have profound effects on the structure and function of FMO3, often leading to Trimethylaminuria (Arseculeratne et al., 2007). Genomic analysis of these polymorphisms indicate key amino acid residues that are integral in the performance of these enzymes when metabolizing specific analytes such as TMA (Motika et al., 2009). Further interpretation of genetic variants has shown specific preferences for xenobiotic substrates providing insight into the preferable substrate models that can be used as a potential therapeutic by reducing expression of FMO3 (Borbas et al., 2006).

Located on the endoplasmic reticulum, FMO’s can oxygenate a wide variety of soft nucleophilic xenobiotics such as heteroatoms containing nitrogen, sulfur, boronic acids, selenium, and phosphorous functional groups (Ziegler, 1993). FMO’s require a FAD prosthetic group and NADPH cofactor for enzymatic activation. Molecular oxygen and NADPH react with FAD to form the activated 4α-hydroperoxy flavin (FAD-OOH) intermediate that reacts with the nucleophilic substrate converting it to its respective oxide form (Phillips and Shephard, 2008). Nucleophilic substrate accessibility to the FMO active site is primarily limited by steric bulk preventing access to the activated 4α-hydroperoxy flavin intermediate (Hines et al., 1994). In Figure 2B TMA can be seen binding in the FMO3 active site with the intermediate NADP^+^ and FAD-OOH. TMA is located at the entrance for nucleophilic substrates to enter the enzymatic cycle and become oxygenated.

Crystallographic data shows coordination of FAD, NADPH, and amino acid Asparagine 91 (Asp 91) as the main focal point at which substrate metabolism occurs. By mapping out the surrounding enzyme binding site *in silico* key structural features can be identified and mechanistic action can be better understood to further aid with drug design (Eswaramoorthy et al., 2006). Crystal structures with methimazole, a known competitive inhibitor (Nace et al., 1997), bound to FMO3 gives further binding information about the possible mechanism of inhibition and help to further drug design of novel therapeutics. Methimazole forms strong pi–pi interactions with the cofactor FAD and is capable of hydrogen boding to the Asp 91 residue in the same location as NADPH and its intermediate NADP^+^ seen in Figure 2D, leading to inhibition of the enzymatic reaction by blocking NADPH from entering the catalytic site (Gao et al., 2018).

Clinical trials using dietary indoles coming from Brussel sprouts have shown efficacy in lowering the relative ratio of TMAO to TMA through inhibition of FMO3. This research discovered that acid condensation products of dietary indoles that can be observed in Figure 2C are the main culprit in the results observed, providing chemical structures with known function and structural characterization shown in Figure 2C (Cashman et al., 1999). Docking studies show that these dietary indoles bind to residues specific for NADPH, similarly to methimazole, and are capable of competitive inhibition of enzymatic activity.

In male diabetic Sprague-Dawley rat models treated with streptozotocin, increased FMO activity and was thus positively correlated with increasing blood glucose levels. However insulin treatment returned FMO activity to normal (non-diabetic) levels. Conversely, insulin treatment had no effect on FMO activity in non-diabetic rats (Borbás et al., 2006). In regards to other forms of FMO, the potentiation of liver injury by thioacetamide, in diabetic rats, due to induced CYP2E1, showed that inhibition of FMO1 using indole-3-carbinol, potentiated liver damage. This study demonstrated that a potential mechanism by which FMO1 inactivates potentially harmful metabolites, and how FMO1 activity increases with increasing blood glucose levels (Wang et al., 2000).

cDNA analysis of biopsied liver tissues from T2D patients have also shown that genes associated with stress defense such as FMO5 and superoxide dismutase 2 are down-regulated when compared to the non-diabetic patients (Takamura et al., 2004). Thus, FMO’s have become an attractive target for drug design and therapeutics due to their ability to metabolize or activate prodrugs and their complex involvement in regulating metabolic function in associated disorders such as T2D and CVD.

Flavin-containing monooxygenase 3’s primary expression has been observed in the liver where it is the primary site for TMA metabolism to TMAO for the purpose of increased solubility and improved renal clearance as observed in Figure 1 (Ayesh and Smith, 1990; Fennema et al., 2016). FMO3 has also demonstrated a significant role in reverse cholesterol transport (RCT), cholesterol absorption in the small intestine, and bile acid formation in FMO3 knockout mice. This result is most likely due to TMAO’s ability to inhibit macrophage RCT contributing to increased plaque formation and an increased risk of CVD (Warrier et al., 2015). In obese-insulin resistant mice, FMO3 was shown to have increased expression contributing to higher levels of circulating TMAO and correlating positively with insulin resistance in human populations during clinical trials (Randrianarisoa et al., 2016).

In patients with chronic kidney disease, elevated levels of TMAO contributed significantly to the development of renal fibrosis resulting in poor mortality outcomes (Tang et al., 2015). These results in connection with other reports demonstrate that TMAO is a significant factor in platelet aggregation, due to promoting Ca^2+^ efflux and vascular smooth muscle cell activation. Further these results also demonstrate the significance that TMAO has on propagating diseases through a variety of metabolic pathways (Zhu et al., 2016).

While FMO3 has garnered the most attention, other FMO enzymes demonstrate potential for therapeutic intervention as well. While FMO5 shows expression in the small intestines, kidneys, lungs, and liver (Overby et al., 1995; Cashman and Zhang, 2006) expression in the small intestines and liver is of the greatest interest for CVD related therapeutics. However, FMO5 differs significantly in substrate catalytic activity and shows low affinity for similar substrates seen in FMO3 (Zhang et al., 2007). While expression of FMO5 in the small intestines is independent of the bacteria present, there is evidence to suggest that a high fat diet does influence FMO5 expression in the small intestines, but this was tissue specific as an increase in FMO5 liver expression was not observed (Scott et al., 2017).

## Conclusion

Even though compounds such as DMB and dietary indoles have shown promise as a therapeutic intervention for CVD and T2D there is still much that can be improved upon in their chemical structures. DMB has only been shown to be effective as a non-lethal inhibitor of bacteria and inhibiting TMAO levels, however it has yet to be demonstrated that any improvements in glucose homeostasis or insulin sensitivity would be observed.

Dietary indoles, while effective at inhibiting FMO3 activity and reducing TMAO levels, (Cashman et al., 1999) have inherent problems with specificity as indoles have shown to be effective serotonin agonists with the significant ability to permeate the blood brain barrier, eliciting potentially harmful psychotropic effects (Chen et al., 2016; Zajdel et al., 2016). Combining the structural features of these chemical compounds into one drug design that is capable of specific sites of action holds promise that significant advancements can be made to attenuate the effects of CVD and T2D in one efficient drug therapy.

No current therapeutics target both bacterial and human metabolism in a way that can reduce systemic inflammation, reorganize cholesterol levels, and improve energy regulation to reduce the effects of aging and dietary overload that is characteristic of patients with CVD and T2D. By combining these targets into one therapeutic, it might be possible to modulate crosstalk in the gut-liver-heart axis, effectively controlling the succeeding cascade of events that play out in rescuing further development of complications that arise in T2D and CVD.

## Author Contributions

IS and NG drafted and finalized the manuscript with support from RA. RA provided the basis for the review and guidance throughout the manuscript development. MG, SY, and JZ involved in subject discussion, formation of material, and manuscript revision. All authors contributed to the article and approved the submitted version.

## Conflict of Interest

The authors declare that the research was conducted in the absence of any commercial or financial relationships that could be construed as a potential conflict of interest.

## References

[B1] Al-Waiz M. Ayesh R. Mitchell S. C. Idle J. R. Smith R. L. (1987). A Genetic polymorphism of the N-oxidation of trimethylamine in humans. *Clin. Pharmacol. Therap.* 42 588–594. 10.1038/clpt.1987.201 3677545

[B2] Arora T. Bäckhed F. (2016). The gut microbiota and metabolic disease: current understanding and future perspectives. *J. Intern. Med.* 280 339–349. 10.1111/joim.12508 27071815

[B3] Arseculeratne G. Wong A. K. C. Goudie D. R. Ferguson J. (2007). Trimethylaminuria (Fish-Odor syndrome): a case report. *Archiv. Dermatol.* 143 81–84. 10.1001/archderm.143.1.81 17224546

[B4] Athyros V. G. Papageorgiou A. A. Athyrou V. V. Demitriadis D. S. Kontopoulos A. G. (2002). Atorvastatin and micronized fenofibrate alone and in combination in Type 2 diabetes with combined hyperlipidemia. *Diabetes Care* 25 1198–1202. 10.2337/diacare.25.7.1198 12087019

[B5] Ayesh R. Smith R. L. (1990). Genetic polymorphism of trimethylamine N-oxidation. *Pharmacol. Therap.* 45 387–401. 10.1016/0163-7258(90)90074-c 2405443

[B6] Bai J. Zheng S. Jiang D. Han T. Li Y. Zhang Y. (2015). Oxidative stress contributes to abnormal glucose metabolism and insulin sensitivity in two hyperlipidemia models. *Intern. J. Clin. Exper. Pathol.* 8 13193–13200. PMC4680463 26722518

[B7] Barrea L. Annunziata G. Muscogiuri G. Di Somma C. Laudisio D. Maisto M. (2018). Trimethylamine-N-oxide (TMAO) as novel potential biomarker of early predictors of metabolic syndrome. *Nutrients* 10:1971 10.3390/nu10121971 PMC6316855 30551613

[B8] Bennett M. R. Sinha S. Owens G. K. (2016). Vascular smooth muscle cells in atherosclerosis. *Circ. Res.* 118 692–702. 26892967 10.1161/CIRCRESAHA.115.306361 PMC4762053

[B9] Bodea S. Funk M. A. Emily P. Balskus L. Catherine L. D. (2016). Molecular basis of C–N bond cleavage by the glycyl radical enzyme Choline Trimethylamine-Lyase. *Cell Chem. Biol.* 23 1206–1216. 10.1016/j.chembiol.2016.07.020 27642068 PMC5493019

[B10] Booth G. L. Kapral M. K. Fung K. Tu J. V. (2006). Relation between age and cardiovascular disease in men and women with diabetes compared with non-diabetic people: a population-based retrospective cohort study. *Lancet* 368 29–36. 10.1016/s0140-6736(06)68967-8 16815377

[B11] Borbás T. Benkõ B. Dalmadi B. Szabó I. Tihanyi K. (2006). Insulin in flavin-containing monooxygenase regulation: flavin-containing monooxygenase and cytochrome P450 activities in experimental diabetes. *Eur. J. Pharmaceut. Sci.* 28 51–58. 10.1016/j.ejps.2005.12.011 16488120

[B12] Borbas T. Zhang J. Cerny M. A. Liko I. Cashman J. R. (2006). Investigation of structure and function of a catalytically efficient variant of the human flavin-containing monooxygenase form 3. *Drug Metab. Dispos.* 34 1995–2002. 10.1124/dmd.106.010827 16985102

[B13] Cashman J. R. Zhang J. (2006). Human flavin-containing monooxygenases. *Ann. Rev. Pharmacol. Toxicol.* 46 65–100. 16402899 10.1146/annurev.pharmtox.46.120604.141043

[B14] Cashman J. R. Xiong Y. Lin J. Verhagen H. van Poppel G. van Bladeren P. J. (1999). In vitro and in vivo inhibition of human flavin-containing monooxygenase form 3 (FMO3) in the presence of dietary indoles. *Biochem. Pharmacol.* 58 1047–1055. 10.1016/s0006-2952(99)00166-5 10509757

[B15] Centers for Disease Control and Prevention (2018). *U.S*. *Diabetes Surveillance System.* Atlanta: Centers for Disease Control and Prevention.

[B16] Chen J. Tao L.-X. Xiao W. Ji S.-S. Wang J.-R. Li X.-W. (2016). Design, synthesis and biological evaluation of novel chiral oxazino-indoles as potential and selective neuroprotective agents against Aβ25–35-induced neuronal damage. *Bioorgan. Med. Chem. Lett.* 26 3765–3769. 10.1016/j.bmcl.2016.05.061 27301369

[B17] Chen S. Henderson A. Petriello M. C. Romano K. A. Gearing M. Miao J. (2019). Trimethylamine N-oxide binds and activates PERK to promote metabolic dysfunction. *Cell Metab.* 30 1141–1151.e5. 31543404 10.1016/j.cmet.2019.08.021

[B18] Craciun S. Marks J. A. Balskus E. P. (2014). Characterization of choline Trimethylamine-Lyase Expands the chemistry of glycyl radical enzymes. *ACS Chem. Biol.* 9 1408–1413. 10.1021/cb500113p 24854437

[B19] Ding H. Triggle C. R. (2005). Endothelial cell dysfunction and the vascular complications associated with type 2 diabetes: assessing the health of the endothelium. *Vasc. Health Risk Manag.* 1 55–71. 10.2147/vhrm.1.1.55.58939 17319098 PMC1993929

[B20] Eswaramoorthy S. Bonanno J. B. Burley S. K. Swaminathan S. (2006). Mechanism of action of a flavin-containing monooxygenase. *Proc. Natl. Acad. Sci. U.S.A.* 103 9832–9837. 16777962 10.1073/pnas.0602398103 PMC1502539

[B21] Fennema D. Phillips I. R. Shephard E. A. (2016). Trimethylamine and Trimethylamine N-Oxide, a Flavin-containing monooxygenase 3 (FMO3)-mediated host-microbiome metabolic axis implicated in health and disease. 44 1839–1850. 10.1124/dmd.116.070615 27190056 PMC5074467

[B22] Ferrannini E. DeFronzo R. A. (2015). Impact of glucose-lowering drugs on cardiovascular disease in type 2 diabetes. *Eur. Heart J.* 36 2288–2296. 10.1093/eurheartj/ehv239 26063450

[B23] Gao C. Catucci G. Gilardi G. Sadeghi S. J. (2018). Binding of methimazole and NADP (H) to human FMO3: in vitro and in silico studies. *Intern. J. Biol. Macromol.* 118 460–468. 10.1016/j.ijbiomac.2018.06.104 29959003

[B24] Hines R. N. Cashman J. R. Philpot R. M. Williams D. E. Ziegler D. M. (1994). The mammalian flavin-containing monooxygenases: molecular characterization and regulation of expression. *Toxicol. Appl. Pharmacol.* 125 1–6. 10.1006/taap.1994.1042 8128486

[B25] Holmes A. J. Chew Y. V. Colakoglu F. Cliff J. B. Klaassens E. Read M. N. (2017). Diet-microbiome interactions in health are controlled by intestinal nitrogen source constraints. *Cell Metab.* 25 140–151. 10.1016/j.cmet.2016.10.021 27889387

[B26] Hosseini-Beheshti E. Grau G. E. R. (2019). Extracellular vesicles and microvascular pathology: decoding the active dialogue. *Microcirculation* 26:e12485. 10.1111/micc.12485 29923276

[B27] Hotamisligil G. S. (2010). Endoplasmic reticulum stress and atherosclerosis. *Nat. Med.* 16:396. 10.1038/nm0410-396 PMC2897068 20376052

[B28] Iglay K. Hannachi H. Joseph Howie P. Xu J. Li X. Engel S. S. (2016). Prevalence and co-prevalence of comorbidities among patients with type 2 diabetes mellitus. *Curr. Med. Res. Opin.* 32 1243–1252. 10.1185/03007995.2016.1168291 26986190

[B29] Janeiro M. Ramírez M. Milagro F. Martínez J. Solas M. (2018). Implication of trimethylamine N-Oxide (TMAO) in disease: potential biomarker or new therapeutic target. *Nutrients* 10:1398 10.3390/nu10101398 PMC6213249 30275434

[B30] Kamo T. Akazawa H. Suda W. Saga-Kamo A. Shimizu Y. Yagi H. (2017). Dysbiosis and compositional alterations with aging in the gut microbiota of patients with heart failure. *PLoS One* 12:e0174099. 10.1371/journal.pone.0174099 28328981 PMC5362204

[B31] Landfald B. Valeur J. Berstad A. Raa J. (2017). Microbial trimethylamine-N-oxide as a disease marker: something fishy? *Microb. Ecol. Health Dis.* 28:1327309 10.1080/16512235.2017.1327309 PMC5444358 28588431

[B32] Lau W. L. Vaziri N. D. (2019). Gut microbial short-chain fatty acids and the risk of diabetes. *Nat. Rev. Nephrol.* 15 389–390. 10.1038/s41581-019-0142-7 30918350

[B33] Leon B. M. Maddox T. M. (2015). Diabetes and cardiovascular disease: epidemiology, biological mechanisms, treatment recommendations and future research. *World J. Diabetes* 6 1246–1258. 10.4239/wjd.v6.i13.1246 26468341 PMC4600176

[B34] Libby P. (2006). Inflammation and cardiovascular disease mechanisms. *Am. J. Clin. Nutr.* 83 456S–460S. 16470012 10.1093/ajcn/83.2.456S

[B35] Liu L. He X. Feng Y. (2019). Coronary heart disease and intestinal microbiota. *Coron. Artery Dis.* 30 384–389. 10.1097/mca.0000000000000758 31276455

[B36] Luedde M. Winkler T. Heinsen F.-A. Rühlemann M. C. Spehlmann M. E. Bajrovic A. (2017). Heart failure is associated with depletion of core intestinal microbiota. *ESC Heart Fail.* 4 282–290. 10.1002/ehf2.12155 28772054 PMC5542738

[B37] Marques F. Z. Nelson E. Chu P.-Y. Horlock D. Fiedler A. Ziemann M. (2017). High-fiber diet and acetate supplementation change the gut microbiota and prevent the development of hypertension and heart failure in hypertensive mice. *Circulation* 135 964–977. 10.1161/circulationaha.116.024545 27927713

[B38] Mitchell S. C. Smith R. L. (2001). Trimethylaminuria: the fish malodor syndrome. *Drug Metab. Disposit.* 29 517–521. 11259343

[B39] Mohammadi A. Najar A. G. Yaghoobi M. M. Jahani Y. Vahabzadeh Z. (2016). Trimethylamine-N-oxide treatment induces changes in the ATP-binding cassette transporter A1 and scavenger receptor A1 in murine macrophage J774A. 1 cells. *Inflammation* 39 393–404. 10.1007/s10753-015-0261-7 26412259

[B40] Motika M. S. Zhang J. Zheng X. Riedler K. Cashman J. R. (2009). Novel variants of the human flavin-containing monooxygenase 3 (FMO3) gene associated with trimethylaminuria. *Mol. Genet. Metab.* 97 128–135. 10.1016/j.ymgme.2009.02.006 19321370 PMC2739593

[B41] Murphy E. A. Velazquez K. T. Herbert K. M. (2015). Influence of high-fat diet on gut microbiota: a driving force for chronic disease risk. *Curr. Opin. Clin. Nutr. Metab. Care* 18 515–520. 10.1097/mco.0000000000000209 26154278 PMC4578152

[B42] Nace C. G. Genter M. B. Sayre L. M. Crofton K. M. (1997). Effect of Methimazole, an FMO Substrate and Competitive Inhibitor, on the Neurotoxicity of 3,3’-Iminodipropionitrile in Male Rats1. *Toxicol. Sci.* 37 131–140. 10.1006/faat.1997.2307 9242586

[B43] Overby L. H. Buckpitt A. R. Lawton M. P. Attaasafoadjei E. Schulze J. Philpot R. M. (1995). Characterization of flavin-containing monooxygenase-5 (FMO5) cloned from human and guinea-pig: evidence that the unique catalytic properties of FMO5 are not confined to the rabbit ortholog. *Archiv. Biochem. Biophys.* 317 275–284. 10.1006/abbi.1995.1163 7872795

[B44] Phillips I. R. Shephard E. A. (2008). Flavin-containing monooxygenases: mutations, disease and drug response. *Trends Pharmacol. Sci.* 29 294–301. 10.1016/j.tips.2008.03.004 18423897

[B45] Pi-Sunyer X. (2014). The look AHEAD trial: a review and discussion of its outcomes. *Curr. Nutr. Rep.* 3 387–391. 10.1007/s13668-014-0099-x 25729633 PMC4339027

[B46] Pluznick J. L. Protzko R. J. Gevorgyan H. Peterlin Z. Sipos A. Han J. (2013). Olfactory receptor responding to gut microbiota-derived signals plays a role in renin secretion and blood pressure regulation. *Proc. Natl. Acad. Sci. U.S.A.* 110 4410–4415. 10.1073/pnas.1215927110 23401498 PMC3600440

[B47] Rachel N. Carmody G. K. Gerber J. M. Luevano D. M. Gatti L. S. Svenson K. L. (2015). Diet dominates host genotype in shaping the murine gut microbiota. *Cell Host Microb.* 17 72–84. 10.1016/j.chom.2014.11.010 PMC4297240 25532804

[B48] Randrianarisoa E. Lehn-Stefan A. Wang X. Hoene M. Peter A. Heinzmann S. S. (2016). Relationship of Serum Trimethylamine N-Oxide (TMAO) Levels with early *Atherosclerosis* in humans. *Sci. Rep.* 6:26745. 10.1038/srep26745 PMC4882652 27228955

[B49] Rath S. Heidrich B. Pieper D. H. Vital M. (2017). Uncovering the trimethylamine-producing bacteria of the human gut microbiota. *Microbiome* 5:54. 10.1186/s40168-017-0271-9 PMC5433236 28506279

[B50] Sandek A. Bjarnason I. Volk H.-D. Crane R. Meddings J. B. Niebauer J. (2012). Studies on bacterial endotoxin and intestinal absorption function in patients with chronic heart failure. *Intern. J. Cardiol.* 157 80–85. 10.1016/j.ijcard.2010.12.016 21190739

[B51] Scott F. Gonzalez Malagon S. G. O’Brien B. A. Fennema D. Veeravalli S. Coveney C. R. (2017). Identification of flavin-containing monooxygenase 5 (FMO5) as a regulator of glucose homeostasis and a potential sensor of gut bacteria. *Drug Metab. Dispos.* 45 982–989. 10.1124/dmd.117.076612 28646079 PMC5539585

[B52] Shih D. M. Zhu W. Schugar R. C. Meng Y. Jia X. Miikeda A. (2019). Genetic deficiency of Flavin-containing monooxygenase 3 (Fmo3) protects against thrombosis but has only a minor effect on plasma lipid levels—brief report. *Thromb. Biol.* 39 1045–1054. 10.1161/atvbaha.119.312592 PMC6531332 31070450

[B53] Silverman M. G. Ference B. A. Im K. Wiviott S. D. Giugliano R. P. Grundy S. M. (2016). Association between lowering LDL-C and cardiovascular risk reduction among different therapeutic interventions: a systematic review and meta-analysis. *JAMA* 316 1289–1297. 10.1001/jama.2016.13985 27673306

[B54] Simon G. L. Gorbach S. L. (1986). The human intestinal microflora. *Digest. Dis. Sci.* 31 147–162. 10.1007/BF01295996 3731990

[B55] Sommer F. Bäckhed F. (2013). The gut microbiota — masters of host development and physiology. *Nat. Rev. Microbiol.* 11 227–238. 10.1038/nrmicro2974 23435359

[B56] Srinivas M. (2017). Cytochrome P450 enzymes, drug transporters and their role in pharmacokinetic drug-drug interactions of Xenobiotics: a comprehensive review. *Open J. Chem.* 3, 1–11.

[B57] Sung M. M. Kim T. T. Denou E. Soltys C.-L. M. Hamza S. M. Byrne N. J. (2017). Improved glucose homeostasis in obese mice treated with resveratrol is associated with alterations in the gut microbiome. *Diabetes* 66 418–425. 10.2337/db16-0680 27903747

[B58] Takamura T. Sakurai M. Ota T. Ando H. Honda M. Kaneko S. (2004). Genes for systemic vascular complications are differentially expressed in the livers of Type 2 diabetic patients. *Diabetologia* 47 638–647. 10.1007/s00125-004-1366-y 15298340

[B59] Tang W. H. Wang Z. Kennedy D. J. Wu Y. Buffa J. A. Agatisa-Boyle B. (2015). Gut microbiota-dependent trimethylamine N-oxide (TMAO) pathway contributes to both development of renal insufficiency and mortality risk in chronic kidney disease. *Circ. Res.* 116 448–455. 10.1161/circresaha.116.305360 25599331 PMC4312512

[B60] Tang W. W. Li D. Y. Hazen S. L. (2019). Dietary metabolism, the gut microbiome, and heart failure. *Nat. Rev. Cardiol.* 16 137–154. 10.1038/s41569-018-0108-7 30410105 PMC6377322

[B61] Tilg H. (2016). A gut feeling about thrombosis. *New Engl. J. Med.* 374 2494–2496. 10.1056/nejmcibr1604458 27332910

[B62] Velasquez M. Ramezani A. Manal A. Raj D. (2016). Trimethylamine N-oxide: the good, the bad and the unknown. *Toxins* 8:326 10.3390/toxins8110326 PMC5127123 27834801

[B63] Wang C. C. L. Hess C. N. Hiatt W. R. Goldfine A. B. (2016). Clinical update: cardiovascular disease in diabetes mellitus. *Circulation* 133 2459–2502. 10.1161/circulationaha.116.022194 27297342 PMC4910510

[B64] Wang T. Shankar K. Ronis M. J. Mehendale H. M. (2000). Potentiation of thioacetamide liver injury in diabetic rats is due to induced CYP2E1. *J. Pharmacol. Exp. Ther.* 294 473–479. 10900221

[B65] Wang Z. Klipfell E. Bennett B. J. Koeth R. Levison B. S. DuGar B. (2011). Gut flora metabolism of phosphatidylcholine promotes cardiovascular disease. *Nature* 472:57. 10.1038/nature09922 21475195 PMC3086762

[B66] Wang Z. Roberts A. B. Buffa J. A. Levison B. S. Zhu W. Org E. (2015). Non-lethal inhibition of gut microbial trimethylamine production for the treatment of atherosclerosis. *Cell* 163 1585–1595. 10.1016/j.cell.2015.11.055 26687352 PMC4871610

[B67] Warrier M. Shih D. M. Burrows A. C. Ferguson D. Gromovsky A. D. Brown A. L. (2015). The TMAO-generating enzyme flavin monooxygenase 3 is a central regulator of cholesterol balance. *Cell Rep.* 10 326–338. 10.1016/j.celrep.2014.12.036 25600868 PMC4501903

[B68] Zajdel P. Marciniec K. Satała G. Canale V. Kos T. Partyka A. (2016). N1-Azinylsulfonyl-1H-indoles: 5-HT6 receptor antagonists with procognitive and antidepressant-like properties. *ACS Med. Chem. Lett.* 7 618–622. 10.1021/acsmedchemlett.6b00056 27326337 PMC4904269

[B69] Zhang J. Cerny M. A. Lawson M. Mosadeghi R. Cashman J. R. (2007). Functional activity of the mouse flavin-containing monooxygenase forms 1, 3, and 5. *J. Biochem. Mol. Toxicol.* 21 206–215. 10.1002/jbt.20176 17721934

[B70] Zhu W. Gregory J. C. Org E. Buffa J. A. Gupta N. Wang Z. (2016). Gut microbial metabolite TMAO enhances platelet hyperreactivity and thrombosis risk. *Cell* 165 111–124. 10.1016/j.cell.2016.02.011 26972052 PMC4862743

[B71] Ziegler D. M. (1993). Recent studies on the structure and function of multisubstrate flavin-containing monooxygenases. *Annu. Rev. Pharmacol. Toxicol.* 33 179–199. 10.1146/annurev.pa.33.040193.001143 8494339

